# A candidate gene study reveals association between a variant of the Peroxisome Proliferator-Activated Receptor Gamma (PPAR-γ) gene and systemic sclerosis

**DOI:** 10.1186/s13075-015-0641-2

**Published:** 2015-05-19

**Authors:** Roberta Goncalves Marangoni, Benjamin D Korman, Yannick Allanore, Philippe Dieude, Loren L Armstrong, Margarita Rzhetskaya, Monique Hinchcliff, Mary Carns, Sofia Podlusky, Sanjiv J Shah, Barbara Ruiz, Eric Hachulla, Kiet Tiev, Jean-Luc Cracowski, John Varga, M Geoffrey Hayes

**Affiliations:** Division of Rheumatology, Department of Medicine, Northwestern University Feinberg School of Medicine, 240 E. Huron Street, McGaw Pavillion M230, Chicago, IL USA; Paris Descartes University, INSERM U1016, Institut Cochin, Sorbonne Paris Cité, Paris, France; Paris Descartes University, Rheumatology A department, Cochin Hospital, APHP, Paris, France; Université Paris 7, INSERM U699, Rhumatologie, Hôpital Bichat, Paris, France; Division of Endocrinology, Metabolism, and Molecular Medicine, Department of Medicine, Northwestern University Feinberg School of Medicine, Chicago, IL USA; Division of Cardiology, Department of Medicine, Northwestern University Feinberg School of Medicine, Chicago, IL USA; Université Lille II, Médecine Interne, Lille, France; Université Pierre et Marie Curie, Service de Médecine Interne, Hôpital Saint Antoine, Paris, France; INSERM CIC3, CHU Grenoble, Grenoble, France; Department of Dermatology, Northwestern University Feinberg School of Medicine, Chicago, IL USA; Center of Genetic Medicine, Northwestern University Feinberg School of Medicine, Chicago, IL USA; Department of Anthropology, Northwestern University, Evanston, IL USA

## Abstract

**Introduction:**

The multifunctional nuclear receptor peroxisome proliferator-activated receptor gamma (PPAR-γ) has potent anti-fibrotic effects, and its expression and activity are impaired in patients with systemic sclerosis (SSc). We investigated PPAR-γ gene (*PPARG*) single nucleotide polymorphisms (SNPs) associated with SSc.

**Methods:**

Tag SNPs spanning *PPARG* were genotyped in a European ancestry US discovery cohort comprising 152 SSc patients and 450 controls, with replication of our top signal in a European cohort (1031 SSc patients and 1014 controls from France). Clinical parameters and disease severity were analyzed to evaluate clinical associations with *PPARG* variants.

**Results:**

In the discovery cohort, a single *PPARG* intronic SNP (rs10865710) was associated with SSc (p = 0.010; odds ratio = 1.52 per C allele, 95% confidence interval 1.10-2.08). This association was replicated in the French validation cohort (p = 0.052; odds ratio = 1.16 per C allele, 95% confidence interval 1.00-1.35). Meta-analysis of both cohorts indicated stronger evidence for association (p = 0.002; odds ratio = 1.22 per C allele, 95% confidence interval 1.07-1.40). The rs10865710 C allele was also associated with pulmonary arterial hypertension in the French SSc cohort (p = 0.002; odds ratio = 2.33 per C allele, 95% confidence interval 1.34-4.03).

**Conclusions:**

A *PPARG* variant is associated with susceptibility to SSc, consistent with a role of PPAR-γ in the pathogenesis of SSc.

**Electronic supplementary material:**

The online version of this article (doi:10.1186/s13075-015-0641-2) contains supplementary material, which is available to authorized users.

## Introduction

Systemic sclerosis (SSc) is a chronic multisystem disease of unknown etiology. The hallmarks of SSc are microvascular dysfunction, autoimmune reactivity and organ fibrosis [1,2]. Systemic sclerosis shows substantial heterogeneity in its clinical manifestations, patterns of organ involvement and natural history [3]. Interstitial lung disease (ILD) and pulmonary arterial hypertension (PAH) are major complications that portend a poor prognosis [2,4,5]. The etiology and pathogenesis of SSc remain poorly understood. Mounting evidence supports the role of genetic factors [6].

Recent studies have established genome-wide significant associations of SSc with the major histocompatibility complex (MHC) region as well as *CD247*, *IRF5*, *IRF8*, *STAT4*, and nominal significance at *GRB10*, *JAZF1*, *KIAA0319L*, *PXK*, *RHOB1*, *RPL41* and *SOX5* [7-10]. Genome-wide association follow-up studies have revealed significant additional association at *ATG5*, *DNASE1L3*, *CSK, SCHIP1-IL12A, IL12RB1, IL12RB2, NFKB1, PPARG, PSD3* and *TNIP1* [11-16]*.* In addition, significant SSc associations have been derived from candidate gene approaches including *TNFSF4, TNFAIP3, BANK1, BLK, CD226, IL2RA, KCNA5* and *TLR2* [3,11]*.* Interestingly, nearly all of these genetic studies implicate genes involved in adaptive or innate immunity that have been also associated with other autoimmune diseases including rheumatoid arthritis, systemic lupus erythematosus, psoriasis, and inflammatory bowel disease [17]. Notwithstanding the prominent fibrotic and vasculopathic features of SSc, genetic studies to date have failed to identify major risk factors related to genes involved in the processes of fibrosis or vascular homeostasis [18].

Progressive fibrosis in the skin and multiple organs contributes to organ failure in SSc, and is ascribed to deregulated fibroblast activation [1]. We have focused our research on the multifunctional nuclear receptor peroxisome proliferator activated receptor-gamma (PPAR-γ). Our findings, subsequently confirmed by others, have delineated unexpected potent anti-fibrotic effects of PPAR-γ *in vitro* and *in vivo* [19-24]. Moreover, we and others have shown that the expression and activity of PPAR-γ are impaired in fibroblasts, lesional skin, and lung tissue from patients with SSc, implicating PPAR-γ as a potentially important factor in pathogenesis [22,25]. Mice deficient in PPAR-γ show increased susceptibility to bleomycin-induced fibrosis [26]. Additionally, serum levels of adiponectin, a direct *PPARG* transcriptional target, are reduced in patients with SSc [27].

In multiple cell types, PPAR-γ is a direct target, and is responsible for the anti-diabetic effects of the glitazone class of drugs [28]. At the cellular level, PPAR-γ regulates adipocyte differentiation, insulin sensitivity, and fat metabolism, and has also been implicated in modulating immunity and inflammation [29,30]. Dysfunction of PPAR-γ is implicated in diverse pathologies including diabetes, glomerulosclerosis, atherosclerosis and pulmonary artery hypertension (PAH) [31].

In light of the potential role of PPAR-γ in pathogenesis of SSc, we hypothesized that genetic variants in the *PPARG* may influence disease susceptibility. Two coding, non-synonymous *PPARG* polymorphisms (rs1801282 (P12A) and rs3856806 (C141T)) have been extensively studied in diabetes, coronary artery disease, the metabolic syndrome, and non-alcoholic fatty liver disease [32-35]. The P12A variant has been associated with increased insulin sensitivity, lower body mass and protection from type 2 diabetes [35], while the C161T variant has been associated with increased body weight [34]. In the present studies we sought to conduct a candidate gene association approach to investigate common variants in the *PPARG* gene with SSc.

## Methods

### Study populations

Patients with SSc were evaluated at the Northwestern Scleroderma Program between 2005 and 2009. Patients and controls were enroled in NUgene, a Northwestern University biobank in which participants gave qualified investigators de-identified access to their retrospective and longitudinal electronic medical record (EMR) information, as well as a blood draw for DNA extraction coded to match their EMR information to conduct genetic studies [36]. These patients self-reported as having European ancestry.

The cohort consisted of 152 SSc patients (53 with diffuse cutaneous SSc (dcSSc), 96 with limited cutaneous SSc (lcSSc), and 2 patients who were unclassified). All patients fulfilled American College of Rheumatology (ACR) criteria for SSc and cutaneous subsets were defined according to the criteria of LeRoy *et al*. [37,38]. Each SSc patient was matched by age, gender, and ancestry to three NUgene biobank controls without evidence of SSc or other clinical autoimmune diseases according to self-report or by ICD-9 codes generated in the course of their clinical care at Northwestern. This study was approved by the institutional review board of Northwestern University, written informed consent was acquired and blood was obtained. The demographic and clinical characteristics of patients with SSc in the discovery and replication sets are presented in Table 1.

**Table 1:** **Clinical characteristics of SSc patients included in the discovery and replication sets**

	**US cohort**	**French cohort**	***P*** **-value**
Patients genotyped, n*	152	1031	
Female	78	86	**0.012**
Diffuse cutaneous systemic sclerosis	35	31	0.578
Limited cutaneous systemic sclerosis	63	63	0.578
Anticentromere antibody-positive	22	41	**<0.001**
Anti-topoisomerase I antibody-positive	23	28	0.860
Interstitial lung disease	57	38	**<0.001**
Pulmonary hypertension	7	7	0.976

*Unless otherwise indicated, values indicate percentages. Bold *P*-values are those that are statistically significant at *P* <0.05 (Student’s *t*-test).

A replication study was performed using a French cohort consisting of 1,031 SSc patients and 1,014 matched controls [39,40]. Both the US discovery and French replication studies included only individuals of European ancestry, defined as having four grandparents of European ancestry.

### Assessment of clinical and laboratory parameters

Clinical and laboratory information obtained at the time of blood sampling included age, gender, race/ethnicity, disease duration (defined as the interval from first SSc-related non-Raynaud event), forced vital capacity (FVC) and carbon monoxide diffusion capacity (DLCO), both expressed as percent of predicted, and high-resolution computerized tomography (HRCT) of the chest. Interstitial lung disease was defined as the presence of pulmonary reticular infiltrates and/or honeycomb cysts on HRCT and/or FVC ≤70% predicted. Screening for pulmonary hypertension (PH) was performed by echo/Doppler and PH was provisionally defined as estimated pulmonary artery systolic pressure ≥40 mmHg; thereafter, the diagnosis was confirmed by right heart catheterization using mean pulmonary artery pressure ≥25 mmHg and capillary pressure <15 mmHg as cut-off values. Anticentromere antibodies (ACA) were detected by indirect immunofluorescence and anti-topoisomerase I antibodies (ATA) were detected by passive immunodiffusion against calf thymus extract (Inova Diagnostics) or by counterimmunoelectrophoresis.

### Selection of single nucleotide polymorphisms (SNPs) genotyped in each cohort

In the US cohort, the tag SNP Picker utility in HapMap [53] was used to select SNPs spanning the 37.5 kb *PPARG* gene and 5 kb up- and downstream to tag common variants in the region [41]. The nine tag SNPs were selected to capture HapMap variants with ≥20% minor allele frequency (MAF) with pairwise *r*^2^ ≤ 0.8 in the European ancestry population (CEU). There was no evidence of long-range linkage disequilibrium (LD) with other genes in the region based on CEU data (Additional file 1). For replication we examined rs10865710 in the French cohort.

### Genotyping

DNA was extracted from blood samples from all NUgene SSc cases and matched controls using the Gentra Autopure LS at the Northwestern University Center for Genetic Medicine Genomics Core Facility. Genotyping was conducted using competitive allele-specific PCR assays (KASP) at KBioscience (Hoddesdon, UK). Cases and controls were randomly distributed across the genotyping plates, and there were no significant plate or batch effects. We removed 19 samples that had four or more (≥25%) SNP assays fail, after which the mean per SNP call rate was 99.3%. Blind duplicates revealed a 99.6% genotyping concordance rate.

In the French cohort, genotyping was performed also using KASP assays at KBioscience. The average genotype completeness was 99% for both the SSc and the control samples. Accuracy was >99%, according to duplicate genotyping of 10% of all samples using the Taqman SNP genotyping assay-allelic discrimination method (Applied Biosystems).

### Statistical analysis

All SNPs were tested for departures from Hardy-Weinberg equilibrium by the chi squared goodness-of-fit test. To test each SNP for association with SSc and subgroups, we computed the overall allelic test of association using the chi squared statistic calculated from two-by-two tables; association statistics and LD patterns were analyzed using Haploview version 4.2 [42]. Meta-analysis of the combined US and French cohorts was performed using the Cochran-Mantel-Haenszel statistical test, and heterogeneity was assessed by calculating I^2^ as described by Higgins *et al*. [43]. We had 80% power to detect associations of odds ratios (ORs) ≥1.3 for SNPs with a risk allele frequency of 20 to 80% in the US cohort at a discovery *P*-value of 0.006 (Bonferroni correction of *P* = 0.05/nine SNPs).

## Results

### Association tests of *PPARG* SNPs with SSc

No significant deviations from Hardy-Weinberg equilibrium (*P* <0.05) were observed for any of the nine SNPs genotyped. In the US population, one SNP (rs10865710) located on the first intron of *PPARG* (Table 2) achieved uncorrected statistical significance (*P* = 0.010; OR = 1.52 (95% CI 1.10, 2.08) per C allele) and narrowly missed our multiple-testing-corrected significance threshold of *P* = 0.006 for association with SSc. To test the robustness of this association, this SNP was then genotyped in the French cohort and found to trend towards association with SSc (*P* = 0.052, OR = 1.16 per C allele, 95% CI 1.00, 1.35). Meta-analysis of the results from the discovery and replication cohorts was performed for SNP rs10865710 in a total combined study population of 1,145 SSc patients and 1,428 controls. The meta-analysis strengthened the association between SSc and rs10865710 (*P* = 0.002, OR = 1.22 per C allele, 95% CI 1.07, 1.40) with no significant evidence for heterogeneity between the two populations (Table 3). We also conducted genotype based tests under dominant and recessive models and found no statistically significant evidence at a Bonferroni-corrected threshold (*P* <0.006) that dominant or recessive models better fit the data than under an additive allelic model.

**Table 2:** **Summary of association of nine** ***PPARG*** **SNPs genotyped in US and French case-control cohorts**

			**US**				**French**		**Meta-analysis**		
**SNP**	**Location**	**Risk/protective alleles**	**Risk allele frequency**		***P*** **-value**	**Odds ratio**		**Odds ratio**		**Odds ratio**	***I*** ^***2***^ **(** ***P*** **-value)**
	**(chr 3)**		**Cases**	**Controls**		**(95% CI)**	***P*** **-value**	**(95% CI)**	***P*** **-value**	**(95% CI)**	
rs2972164	12309416	T/C	0.51	0.48	0.349	1.17					
						(0.90, 1.53)					
rs7620165	12319441	G/A	0.36	0.35	0.679	1.06					
						(0.80, 1.39)					
rs10865710	12328198	C/G	0.81	0.73	**0.010**	1.52	0.052	1.16	**0.002**	1.22	33.6
						(1.10, 2.08)		(1.00, 1.35)		(1.07, 1.40)	(0.134)
rs10510418	12363563	C/A	0.35	0.34	0.592	1.07					
						(0.82, 1.41)					
rs4135247	12371588	G/A	0.45	0.39	0.092	1.25					
						(0.96, 1.63)					
rs2959273	12417731	C/T	0.62	0.62	0.999	1.00					
						(0.76, 1.30)					
rs1151999	12422153	C/A	0.52	0.49	0.353	1.13					
						(0.87, 1.46)					
rs709151	12429999	G/A	0.66	0.64	0.539	1.09					
						(0.82, 1.43)					
rs1175540	12440243	C/A	0.65	0.64	0.736	1.04					
						(0.79, 1.37)					

SNP, single-nucleotide polymorphism; chr, chromosome; SSc, systemic sclerosis; *I* ^*2*^, Higgins *et al*. test for heterogeneity. Bold indicates statistically significant *P*-values.

**Table 3:** **Meta-analysis of rs10865710 association with SSc subtypes in the US and French cohorts**

		**Overall**		**lcSSc**		**dcSSc**	
		**Cases**	**Controls**	**Cases**	**Controls**	**Cases**	**Controls**
US	Number	152	450	96	450	53	450
	C, %	80.6	73.2	83.3	73.2	75.5	73.2
	G, %	19.4	26.8	16.7	26.8	24.5	26.8
	*P*-value	**0.010**		**0.004**		0.619	
	Odds ratio (95% CI)	1.52 (1.10, 2.08)		1.78 (1.19, 2.65)		1.12 (0.71, 1.78)	
French	Number	993	978	632	978	303	978
	C, %	79.0	76.4	77.6	76.4	82.3	76.4
	G, %	21.0	23.6	22.4	23.6	17.7	23.6
	*P*-value	0.052		0.438		**0.002**	
	Odds ratio (95% CI)	1.16 (1.00, 1.35)		1.07 (0.90, 1.27)		1.43 (1.14, 1.81)	
Meta-analysis	Number	1145	1428	728	1428	356	1428
Fixed effects	*P*-value	**0.002**		**0.028**		**0.002**	
	Odds ratio (95% CI)	1.22 (1.07, 1.40)		1.16 (1.00, 1.36)		1.37 (1.11, 1.69)	
	*I* ^*2*^	33.6		41.5		0	
	*P*-value	0.134		0.024		0.376	
Random-effects	*P*-value			0.432			

SSc = systemic sclerosis; lcSSc = limited cutaneous SSc; dcSSc = diffuse cutaneous SSc; OR = odds ratio; 95% CI = 95% confidence interval. ORs are in reference to the risk allele. *I* ^*2*^ = Higgins et al. test for heterogeneity. Bold indicates statistically significant *P*-values.

### Association with disease subtypes and clinical characteristics

Significant associations of rs10865710 were observed with both the lcSSc and dcSSc when each subtype was compared to controls. In the US cohort, this SNP was associated with lcSSc (*P* = 0.004, OR = 1.78 per C allele, 95% CI 1.19, 2.65), whereas in the French cohort this SNP was associated with dcSSc (*P* = 0.002, OR = 1.43 per C allele, 95% CI 1.14, 1.81) (Table 3; Additional file 2). Meta-analyses of the US and French results revealed that both lcSSc (*P* = 0.028, OR = 1.16 per C allele, 95% CI 1.00, 1.36) and dcSSc (*P* = 0.002, OR = 1.37 per C allele, 95% CI 1.11, 1.69) were associated with this SNP. Significant heterogeneity between the two populations was observed for lcSSc, and after adjusting for this the association between rs10865710 was no longer significant (Table 3). Testing for associations with SSc-specific autoantibodies and clinical manifestations (ILD, PH) within cases alone showed an association of SNP rs10865710 with PH in the French cohort (*P* = 0.002, OR = 2.33 per C allele, 95% CI 1.34, 4.03), and in the US-French meta-analysis (*P* = 0.001, OR = 2.38 per C allele, 95% CI 1.40, 4.03) (Additional file 3).

## Discussion

We report an association between SSc and genetic variation within the *PPARG* gene. This novel finding along with a recent report of association of SSc with an SNP 70 kb upstream of the *PPARG* locus add to the mounting evidence of the importance of PPAR-γ in SSc [13]. PPAR-γ is a multi-functional nuclear receptor implicated in a diverse range of metabolic and degenerative diseases, and increasingly in SSc and other fibrotic disorders [26]. This case-control study provides evidence supporting our hypothesis for an association between common variants in the *PPARG* locus and SSc in European ancestry populations. The *PPARG* rs10865710-C susceptibility allele was associated with SSc in the US cohort, trended towards association in the French cohort, and was significantly associated in a combined sample of 1,145 patients and 1,428 controls. The C allele was associated with a 1.22-fold increase in the odds of susceptibility to SSc over the G allele in the combined meta-analysis, and the population-specific ORs ranged from 1.16 (French) to 1.52 (US).

The association signal observed between the rs10865710-C and SSc is most likely not derived from the coding variants rs1801282 (P12A) and rs3856806 (C161T) previously associated with diabetes and coronary artery disease [32,34,35] because they are not in strong LD with rs10865710 (*r*^2^ = 0.31 and 0.14 for P12A and C161T, respectively). Given the limited power of our study we were not able to test for associations at these or any other SNPs with MAF <20% as observed for these two coding variants. There is also no evidence of long-range LD between rs10865710 and other nearby loci; LD between rs10865710 and all chromosome-3 SNPs with MAF ≥5% from HapMap phase-II CEU samples showed no SNP >150 kb from rs10865710 with *r*^2^ ≥ 0.8. This demonstrates that the effect of the associated SNP is unlikely to be modulating any gene other than *PPARG.*

It is noteworthy that the rs10865710 *PPARG* association with SSc has not been found in genome-wide association studies (GWAS0 [7-10]. This may be due to the low effect size limiting the power to achieve genome-wide significance. In fact, a recent GWAS follow-up study found an association of rs310746, an intergenic SNP 70 kb upstream of the PPARG locus, with SSc nearly reaching genome-wide significance [13] and larger meta-analyses will be required to elucidate this. This effect is likely to be independent of the association between SSc and rs10865710 given the low LD between these two SNPs (HapMap CEU *r*^2^ = 0.02).

The SSc-associated SNP rs10865710 is located within the first intron of *PPARG* <1 kb from the second exon. As a tag SNP, it is unclear whether the functional relevance of rs10865710 in SSc is derived from this SNP or another SNP that is in LD. Review of data from the ENCODE project and 1000 Genomes Project using HaploReg did not identify any study analyzing the functional relevance of *PPARG* variation in cell lines relevant to SSc pathogenesis [44,45]. More generally, these databases reveal that the region tagged by rs10865710 alters the binding motif for transcription factor Pou3f2. Moreover, several SNPs in strong LD with rs10865710 (*r*^2^ > 0.9) are located in binding regions for important regulatory proteins including *STAT3* (rs17036242) and glucocorticoid receptor *GR* (rs13433696). While these potential mechanisms are intriguing, there is no evidence that rs10865710 or other SNPs in strong LD have any direct effect on *PPARG* mRNA expression levels. Nor is there evidence to date that this intronic SNP is important for alternative mRNA splicing, epigenetic modification of supercoiled DNA, or miRNA binding. Resequencing of the LD block tagged by rs10865710 may be needed in order to identify one or more SSc causal variants. As in the present studies only common variants were assayed, it remains possible that rare variants with a large effect could be in LD with rs10865710 and impact gene/protein expression in a direct fashion. Deep resequencing at the *PPARG* locus in SSc patients will therefore be required to define potentially causal variants.

A survey of SCANdb [46] reveals that rs10865710 is an eQTL for *GOLGA3* (*P* = 3 × 10^−5^) and *AKAP8* (*P* = 1 × 10^−4^) in European ancestry populations. *GOLGA3* is a golgi apparatus gene which is ubiquitously expressed and does not appear to have any specificity that would make it likely to contribute to SSc pathogenesis based on current knowledge [47]. *AKAP8* is a scaffold protein involved in protein kinase A (PKA) signaling, and although unlikely to be important in SSc, one could hypothesize that the role of this molecule in dendritic cell antigen presentation could be of importance, given that plasmacytoid dendritic cells are considered important in SSc pathogenesis [48,49].

While the US and French cohorts individually found contrasting SSc subtype associations with rs10865710, the combined analysis showed association with both limited and diffuse SSc subtypes. It is unlikely that differences in clinical assessment account for this discrepancy as both cohorts used the same criteria to define SSc subtypes. The fact that both subtypes are significant in the meta-analysis suggests that the contrasting associations may be due to stochastic variability due to small sample sizes of each individual cohort, particularly in the US cohort.

The overall lack of association of rs10865710 with SSc-specific antibodies is not unexpected as *PPARG* has no major effect on adaptive immunity. The ability to detect association with ILD and especially PH were quite likely limited by our small sample size of SSc cases measured for these phenotypes. However, the French cohort and the meta-analysis did have enough patients with right heart catheterization proven PH to demonstrate an association. PPAR-γ has previously been implicated in PH through studies of mice with targeted deletion of PPAR-γ in arterial smooth muscle cells that spontaneously develop PH [50]. Given other multiple studies suggesting an important role of PPAR-γ in pulmonary hypertension [51,52], it is intriguing as to whether the associated SNP may be a risk factor for severe vasculopathy and may warrant further study in larger populations of patients with PH.

A limitation of this study is that the participants in the US-European ancestry cohort self-identified as European ancestry, so it is possible that population genetic substructure could be the underlying cause of the observed association. To correct for this possibility would require genotyping of approximately 100 ancestry informative markers, which was not feasible within the limited scope of this study.

## Conclusion

In conclusion, the present studies provide evidence for association of SSc with an intronic *PPARG* SNP the function of which appears to be independent of known coding variants. These results combined with findings from previous *in vitro* and *in vivo* studies, provide support for the potential role of PPAR-γ in the pathogenesis of fibrotic and vascular complications of SSc. The observations suggest that pharmacological regulation of PPAR-γ expression or activity might represent an innovative approach for the treatment of patients with SSc.

## Additional files

Additional file 1:
**Linkage disequilibrium map for** ***PPARG*** **region.** Linkage disequilibrium (D’) in European ancestry population (CEU) individuals across the entire PPARG locus, green line indicates area in which SNPs were genotyped for the present study. The green dot indicates the location of the systemic sclerosis SSc associated variant rs10865710. Additional file 2:
**Meta-analysis of all single nucleotide polymorphisms (SNPs) genotyped in limited cutaneous systemic sclerosis (lcSSc) and diffuse cutaneous systemic sclerosis (dcSSc) in the US and French cohorts.** Genotype prevalence of all SNPs genotyped separated by disease subtype (lcSSc and dcSSc) including meta-analysis for the associated SNP rs10865710. Additional file 3:
**Association of rs10865710 with systemic sclerosis (SSc) autoantibodies and clinical manifestations.** Genotype prevalence of the associated single nucleotide polymorphism (SNP) rs10865710 separated by autoantibodies (anticentromere and antitopoisomerase I) and presence/absence of interstitial lung disease and pulmonary hypertension.

## Abbreviations

ACA: anticentromere antibodies
ACR: American College of Rheumatology
ATA: anti-topoisomerase I antibodies
CEU: European ancestry population
dcSSc: diffuse cutaneous systemic sclerosis
DLCO: carbon monoxide diffusion capacity
EMR: electronic medical record
FVC: forced vital capacity
GWAS: genome-wide association study
HRCT: high-resolution computerized tomography
ILD: interstitial lung disease
lcSSc: limited cutaneous systemic sclerosis
LD: linkage disequilibrium
MAF: minor allele frequency
MHC: major histocompatibility complex
miRNA: microribonucleic acid
mRNA: messenger ribonucleic acid
OR: odds ratio
PAH: pulmonary arterial hypertension
PCR: polymerase chain reaction
PPAR-γ: peroxisome proliferator activated receptor-gamma
SNP: single nucleotide polymorphism
SSc: systemic sclerosis
