# Supplementary material for: A candidate gene study reveals association between a variant of the Peroxisome Proliferator-Activated Receptor Gamma (PPAR-γ) gene and systemic sclerosis
Source: Arthritis Res Ther. 2015 May 19;17(1):128. doi: 10.1186/s13075-015-0641-2 (PMC4437446; doi:10.1186/s13075-015-0641-2)
Supplement: Additional file 1: — Linkage disequilibrium map for PPARG region. Linkage disequilibrium (D’) in European ancestry population (CEU) individuals across the entire PPARG locus, green line indicates area in which SNPs were genotyped for the present study. The green dot indicates the location of the systemic sclerosis SSc associated variant rs10865710. [file 13075_2015_641_MOESM1_ESM.doc]

**Additional file 1.** Linkage disequilibrium (D’) in CEU individuals across the entire PPARG locus, green line indicates area in which SNPs were genotyped for the present study. The green dot indicates the location of the SSc associated variant rs10865710.

**
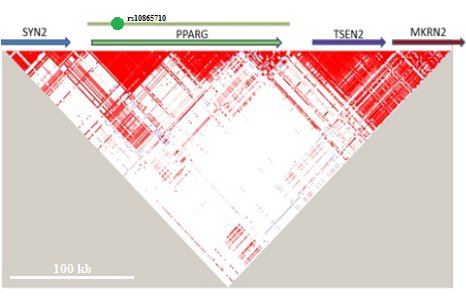
**
