# Supplementary material for: A candidate gene study reveals association between a variant of the Peroxisome Proliferator-Activated Receptor Gamma (PPAR-γ) gene and systemic sclerosis
Source: Arthritis Res Ther. 2015 May 19;17(1):128. doi: 10.1186/s13075-015-0641-2 (PMC4437446; doi:10.1186/s13075-015-0641-2)
Supplement: Additional file 2: — Meta-analysis of all single nucleotide polymorphisms (SNPs) genotyped in limited cutaneous systemic sclerosis (lcSSc) and diffuse cutaneous systemic sclerosis (dcSSc) in the US and French cohorts. Genotype prevalence of all SNPs genotyped separated by disease subtype (lcSSc and dcSSc) including meta-analysis for the associated SNP rs10865710. [file 13075_2015_641_MOESM2_ESM.doc]

**Additional file 2.** Meta-analysis of all SNPs genotyped in lcSSc and dcSSc in the US and French Cohorts

|  |  | **US** | | | | **French** | | | | **Meta-analysis** |  |
| --- | --- | --- | --- | --- | --- | --- | --- | --- | --- | --- | --- |
| SNP | Risk/  Protective  alleles | Risk allele frequency |  | p-value | OR (95% C.I.) | Risk allele frequency |  | p-value | OR (95% C.I.) | p-value OR (95% C.I.) | |
|  |  | lcSSc dcSSc  n=96 n=53 | controls  n=450 | lcSSc dcSSc | lcSSc dcSSc | lcSSc dcSSc  n=632 n= 303 | controls  n=978 | lcSSc dcSSc | lcSSc dcSSc | lcSSc dcSSc | lcSSc dcSSc |
| rs2972164 | T/C | 0.52 0.52 | 0.48 | 0.399 0.592 | 1.14 (0.84-1.56) 1.11 (0.75-1.66) |  |  |  |  |  |  |
| rs7620165 | G/A | 0.36 0.36 | 0.35 | 0.650 0.946 | 1.08 (0.78-1.49) 0.98 (0.65-1.50) |  |  |  |  |  |  |
| rs10865710 | C/G | 0.83 0.75 | 0.73 | **0.004** 0.617 | 1.78 (1.19-2.65) 1.12 (0.71-1.78) | 0.78 0.83 | 0.76 | 0.438 **0.002** | 1.07 (0.90-1.27) 1.43 (1.14-1.81) | **0.028** **0.002** | 1.16 (1.00-1.36) 1.37 (1.11-1.69) |
| rs10510418 | C/A | 0.35 0.37 | 0.34 | 0.759 0.719 | 1.05 (0.76-1.46) 1.08 (0.72-1.63) |  |  |  |  |  |  |
| rs4135247 | G/A | 0.48 0.38 | 0.39 | 0.025 0.976 | 1.43 (1.04-1.95) 1.01 (0.67-1.51) |  |  |  |  |  |  |
| rs2959273 | C/T | 0.64 0.60 | 0.62 | 0.747 0.943 | 1.05 (0.76-1.45) 0.98 (0.66-1.48) |  |  |  |  |  |  |
| rs1151999 | C/A | 0.56 0.54 | 0.49 | 0.134 0.837 | 1.27 (0.93-1.73) 0.96 (0.64-1.42) |  |  |  |  |  |  |
| rs709151 | G/A | 0.32 0.37 | 0.64 | 0.278 0.858 | 1.20 (0.86-1.67) 1.04 (0.68-1.58) |  |  |  |  |  |  |
| rs1175540 | C/A | 0.67 0.61 | 0.64 | 0.317 0.837 | 1.18(0.85-1.64) 0.96 (0.63-1.44) |  |  |  |  |  |  |

* SNP = single-nucleotide polymorphism; MAF = minor allele frequency; SSc = systemic sclerosis; lcSSc = limited cutaneous SSc; dcSSc = diffuse cutaneous SSc; OR = odds ratio; 95% C.I. = 95% confidence interval. ORs are in reference to the reference alleles
