# Supplementary material for: A candidate gene study reveals association between a variant of the Peroxisome Proliferator-Activated Receptor Gamma (PPAR-γ) gene and systemic sclerosis
Source: Arthritis Res Ther. 2015 May 19;17(1):128. doi: 10.1186/s13075-015-0641-2 (PMC4437446; doi:10.1186/s13075-015-0641-2)
Supplement: Additional file 3: — Association of rs10865710 with systemic sclerosis (SSc) autoantibodies and clinical manifestations. Genotype prevalence of the associated single nucleotide polymorphism (SNP) rs10865710 separated by autoantibodies (anticentromere and antitopoisomerase I) and presence/absence of interstitial lung disease and pulmonary hypertension. [file 13075_2015_641_MOESM3_ESM.doc]

**Additional file 3.** Association of rs10865710 with SSc autoantibodies and clinical manifestations

|  |  | ATA | | ACA | | ILD | | PH | |
| --- | --- | --- | --- | --- | --- | --- | --- | --- | --- |
|  |  | positive | negative | positive | negative | present | absent | present | absent |
| US | n | 32 | 106 | 31 | 110 | 63 | 47 | 4 | 57 |
|  | C (%) | 76.6 | 79.7 | 82.3 | 78.6 | 81 | 84 | 91.7 | 80 |
|  | G (%) | 23.4 | 20.3 | 17.7 | 21.4 | 19 | 16 | 8.30 | 20 |
|  | p-value | 0.587 | | 0.533 | | 0.553 | | 0.165 | |
|  | OR (95% C.I.) | 0.83 (0.43 - 1.64) | | 1.27 (0.61 - 2.63) | | 0.81 (0.40 - 1.67) | | 2.75 (0.66-11.48) | |
|  |  |  |  |  |  |  |  |  |  |
| French | n | 237 | 756 | 364 | 629 | 340 | 653 | 66 | 927 |
|  | C (%) | 79.3 | 78.9 | 78.4 | 79.3 | 80.6 | 78.2 | 89.4 | 78.4 |
|  | G (%) | 20.7 | 21.1 | 21.6 | 20.7 | 19.4 | 21.8 | 10.6 | 21.6 |
|  | p-value | 0.844 | | 0.636 | | 0.211 | | **0.002** | |
|  | OR (95% C.I.) | 1.03 (0.79 - 1.32) | | 0.95 (0.76 - 1.19) | | 1.16 (0.92 - 1.45) | | 2.33 (1.34-4.03) | |
|  |  |  |  |  |  |  |  |  |  |
| Meta-analysis | n | 269 | 862 | 395 | 739 | 403 | 700 | 70 | 984 |
|  | p-value | 0.499 | | 0.603 | | 0.157 | | **0.001** | |
|  | OR (95% C.I.) | 1.00 (0.79 - 1.27) | | 1.03 (0.83 - 1.27) | | 1.12 (0.90 - 1.39) | | 2.38 (1.40-4.03) | |
|  |  |  |  |  |  |  |  |  |  |

* SSc = systemic sclerosis; ATA = anti-topoisomerase I antibody; ACA = anticentromere antibody; ILD = interstitial lung disease; PH = pulmonary hypertension; OR = odds ratio; 95% C.I. = 95% confidence interval. Odds ratios (OR) are in reference to the C risk allele.
